# Monkeypox in a Non-endemic Setting: Challenges in the Diagnosis and Management of an Atypical Case

**DOI:** 10.7759/cureus.79547

**Published:** 2025-02-24

**Authors:** Yves N Mrad, Kalpana Jain, Jogi Vikas, Axel B Lichtenberg, Ali Z Ansari, Nilay Bhatt, Shivam Gupta

**Affiliations:** 1 Department of Internal Medicine, HCA Houston Healthcare Clear Lake, Webster, USA; 2 Department of Internal Medicine, William Carey University College of Osteopathic Medicine, Hattiesburg, USA

**Keywords:** fever of obscure origin, monkeypox diagnosis, monkeypox symptoms, monkeypox treatment, monkeypox virus, non-endemic region, oral dysphagia, orthopoxvirus, viral lymphadenopathy, zoonotic infections

## Abstract

Monkeypox is a zoonotic viral disease caused by the monkeypox virus, an Orthopoxvirus closely related to smallpox. While historically confined to endemic regions in Africa, recent outbreaks have demonstrated its capacity to spread globally, posing significant diagnostic and therapeutic challenges. We present the case of a 28-year-old previously healthy Caucasian male who repeatedly sought care in the emergency department (ED) for nonspecific flu-like symptoms, including fever, lymphadenopathy, dysphagia, and a progressive vesiculopustular rash. The initial lack of a clear clinical picture led to multiple misdiagnoses and treatments for presumed bacterial infections. Despite multiple evaluations and initial misdiagnoses, the persistence and progression of symptoms - including worsening lymphadenopathy, dysphagia, and a vesiculopustular rash with umbilicated lesions - raised concern for an Orthopoxvirus infection. Given the patient's history of intimate contact with a male partner who had recently traveled internationally, monkeypox became a key diagnostic consideration. Other possible differential diagnoses included bacterial tonsillitis, peritonsillar abscess, syphilis, varicella, and other sexually transmitted infections. This evolving clinical picture prompted expedited non-variola Orthopoxvirus DNA polymerase chain reaction (PCR) testing, which ultimately confirmed the diagnosis.

This report highlights the diagnostic challenges posed by monkeypox in non-endemic settings, where healthcare providers may have limited familiarity with the disease. It demonstrates the need for heightened clinical suspicion in patients with compatible symptoms, especially during outbreak periods. The patient’s subsequent treatment with tecovirimat demonstrated its potential effectiveness in mitigating symptoms and improving outcomes when initiated promptly. Additionally, this report emphasizes the crucial role of public health measures, including robust surveillance systems such as enhanced case reporting, rapid diagnostic testing networks, and contact tracing programs, alongside healthcare provider education and vaccination strategies, in combating the spread of this reemerging infectious disease.

## Introduction

Monkeypox is a zoonotic viral disease caused by the monkeypox virus, a double-stranded DNA virus belonging to the Orthopoxvirus genus, which also includes variola (smallpox), cowpox, and vaccinia viruses [1]. The virus was first identified in 1958 during an outbreak among research monkeys, which gave the disease its name [2]. However, rodents are now understood to be the primary reservoir. Human cases were first documented in the Democratic Republic of Congo (DRC) and have since been endemic to parts of Central and West Africa, where transmission typically occurs through direct contact with infected animals or contaminated materials [3]. Exposure to bushmeat, bites, or scratches from infected wildlife has historically been the primary mode of zoonotic transmission [4].

Clinically, monkeypox is characterized by an incubation period lasting several days, followed by a febrile prodrome with symptoms such as fever, headache, lymphadenopathy, back pain, and fatigue. Within a few days of fever onset, a vesiculopustular rash develops, often starting on the face and extremities before spreading centrifugally to the rest of the body, including mucous membranes [1]. This rash progresses through distinct stages - macular, papular, vesicular, pustular, and finally, crusting - over several weeks. While the rash is a key diagnostic feature, its resemblance to smallpox lesions can complicate the diagnosis, particularly in endemic regions where other febrile illnesses with similar presentations are common [1].

In recent years, monkeypox has emerged as a global public health challenge, with cases reported in non-endemic regions across multiple continents [5]. This expanded geographic distribution reflects significant changes in the virus’s transmission dynamics and highlights the impact of increased global travel, human-animal interaction, and diminished immunity due to the cessation of smallpox vaccination programs. Traditionally, monkeypox was believed to spread primarily through direct contact with infected animals or individuals. However, recent outbreaks have highlighted the increasing role of person-to-person transmission, including respiratory droplets, direct skin contact, and intimate contact during sexual activity [6].

These shifts in transmission pathways have also been associated with atypical clinical presentations, particularly in high-risk populations such as men who have sex with men (MSM) [7]. Unlike the classic rash that predominantly affects the face and extremities, recent cases have reported localized lesions in the anogenital region, along with symptoms such as proctitis, rectal pain, penile edema, and necrotic skin lesions. Other unusual features, including tonsillar swelling with dysphagia and odynophagia, have added further complexity to the clinical spectrum [8]. These atypical presentations can delay diagnosis and increase the risk of further transmission, particularly in non-endemic regions where healthcare providers may have limited experience with monkeypox.

## Case presentation

A 28-year-old previously healthy Caucasian male presented to the emergency department (ED) with complaints of a sore throat, subjective chills, and fever. He described the symptoms as typical of prior viral illnesses and denied gastrointestinal or genitourinary complaints. Notably, he reported difficulty swallowing and mild fatigue. A throat swab for bacterial infections, including Group A Streptococcus, was negative, and the patient was discharged with instructions for symptomatic management of presumed viral pharyngitis.

**Figure 1:**
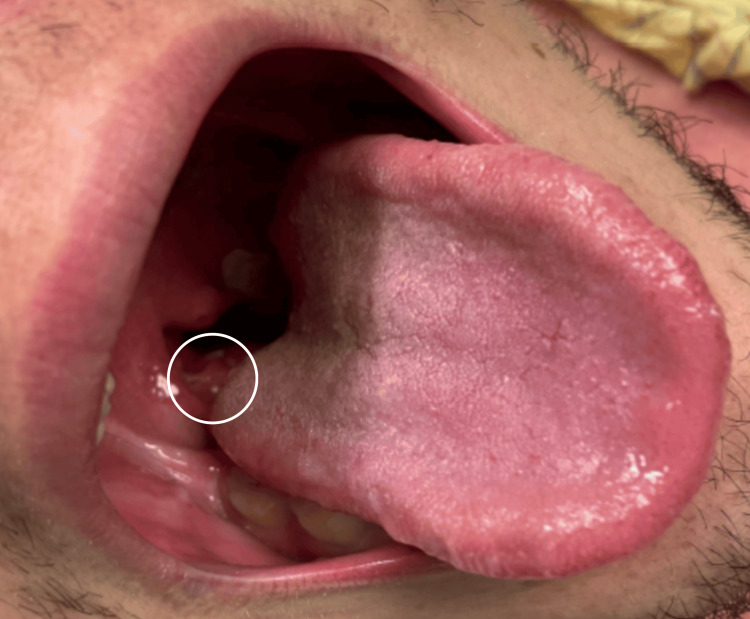
Enlargement of the right tonsil (indicated by the white circle)

**Figure 2:**
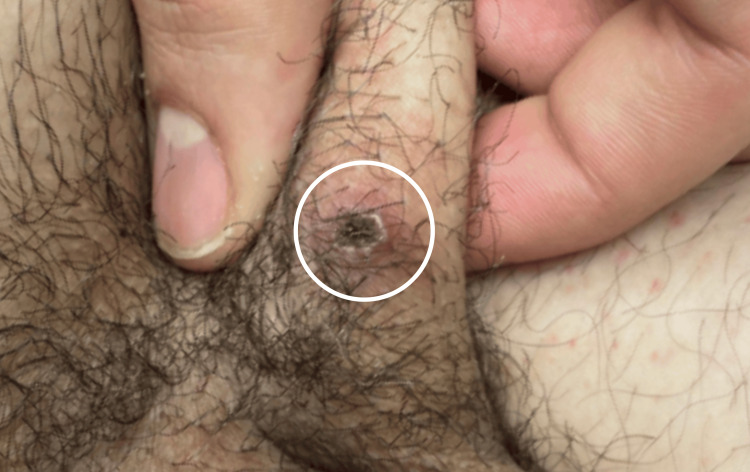
Lesion in the left groin (indicated by the white circle)

**Figure 3:**
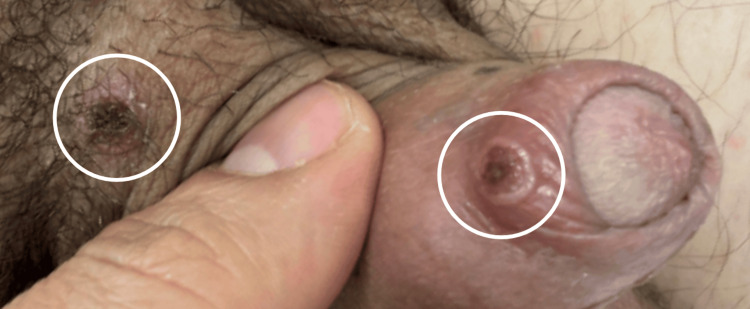
Lesions at the base and foreskin of the penis (highlighted by the white circles)

**Figure 4:**
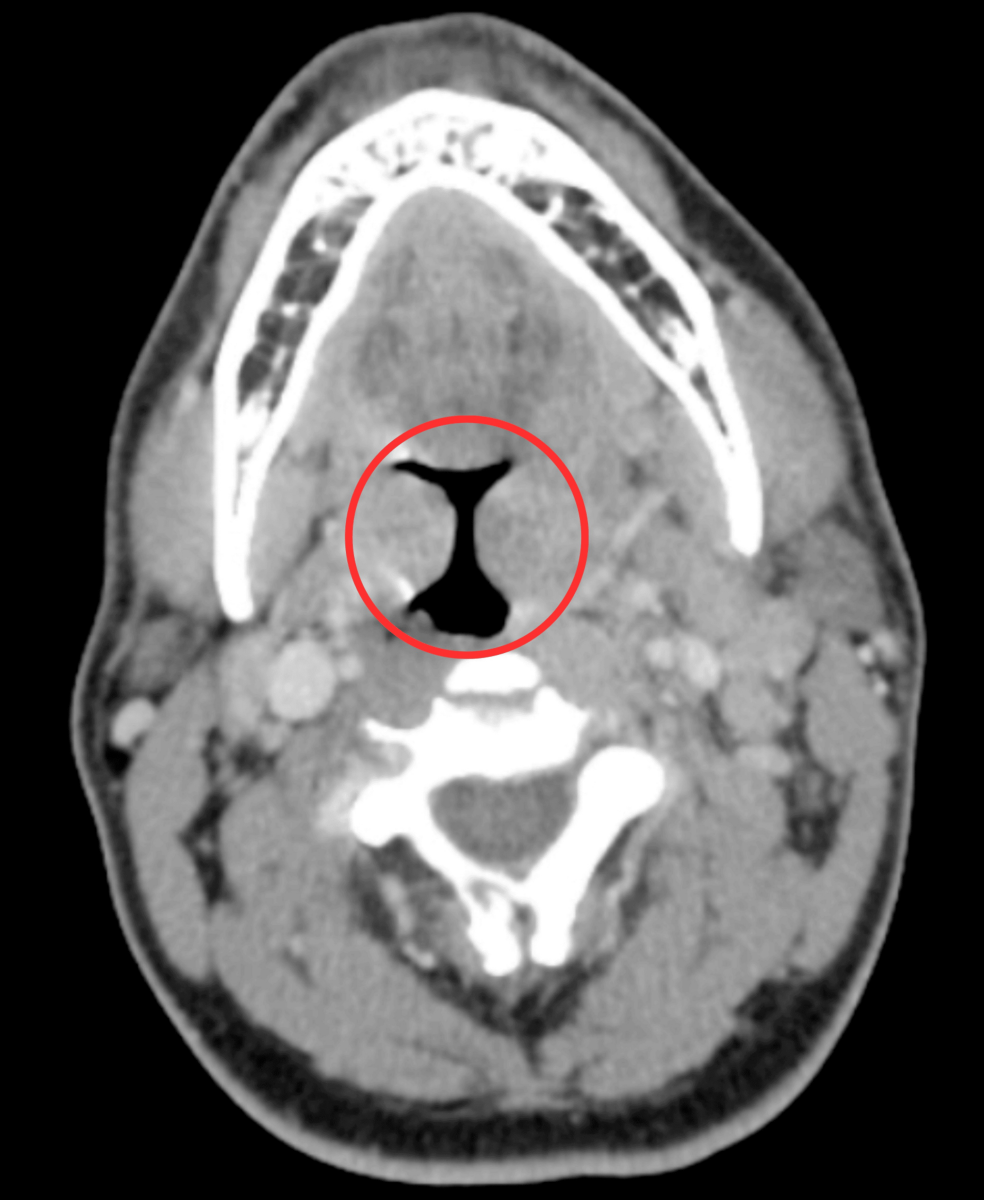
CT scan showing enlarged and heterogeneous bilateral palatine tonsils (indicated by the red circle), suggestive of acute tonsillitis CT: computed tomography

He returned to the ED the next day with worsening symptoms, including right submandibular and tonsillar swelling (Figure 1), along with the development of blistering lesions in the groin area (Figures 2, 3). At this stage, septic peritonsillar abscess was suspected due to the clinical presentation of significant tonsillar swelling and localized pain. The patient was treated empirically with intravenous ampicillin/sulbactam and vancomycin while awaiting imaging and laboratory results. A CT scan of the neck revealed acute tonsillitis without abscess formation (Figure 4). The groin lesions were initially attributed to friction or a superficial fungal infection. Given the evolving presentation, histopathological examination was considered; however, it was not performed as the clinical suspicion for an Orthopoxvirus infection was high, and non-variola orthopoxvirus DNA polymerase chain reaction (PCR) testing was prioritized for definitive diagnosis. Screening for sexually transmitted infections (STIs), including HIV, syphilis, and gonorrhea, was negative.

Non-variola Orthopoxvirus DNA PCR and additional diagnostic tests were ordered but were pending at discharge. The PCR sample was obtained via swabs of the pustular skin lesions and ulcerated groin lesions, following Centers for Disease Control and Prevention (CDC) guidelines for monkeypox testing. The patient was prescribed oral doxycycline and azithromycin as a precautionary measure for possible bacterial coinfections.

Two days later, the patient presented to the ED for a third time with worsening symptoms. He reported severe left submandibular lymphadenopathy, progressive dysphagia, a diffuse rash on the upper extremities and chest, and non-healing groin lesions. The rash, initially localized, had evolved into pustules with surrounding erythema, while the groin lesions exhibited central necrosis and scabbing. The patient described significant groin pain that hindered ambulation and sitting. He also reported new-onset fatigue and anorexia. During this visit, the patient disclosed a history of oral and anal sex with male partners, one of whom had recently traveled internationally.

Physical examination revealed erythematous tonsils, significant left submandibular swelling, and a widespread pustular rash with scattered vesicles. Several pustules on the chest displayed an umbilicated appearance, characteristic of Orthopoxvirus infections. The groin lesions had progressed, displaying central ulceration and scabbing. Despite the severity of the skin findings, vital signs were within normal limits, and laboratory results showed no leukocytosis or systemic inflammatory markers. Rapid plasma reagin (RPR), monospot, and gonorrhea PCR tests were negative. Given the constellation of symptoms, the clinical team expedited non-variola orthopoxvirus DNA PCR testing, suspecting monkeypox as the underlying cause.

Two days later, the non-variola orthopoxvirus DNA PCR results returned positive, confirming the diagnosis of monkeypox. The CDC was promptly notified. The patient was initiated on tecovirimat (600 mg orally every 12 hours) for 14 days as a specific antiviral therapy. Empiric antibiotics were discontinued, and a short course of methylprednisolone was tapered as his symptoms improved. Supportive care included pain management with acetaminophen and applying topical emollients to soothe the lesions. The patient was hospitalized for one week and demonstrated steady clinical improvement. At discharge, he was advised to remain isolated and avoid close contact until all lesions had crusted and resolved completely. At his one-week follow-up appointment, the patient reported complete resolution of his symptoms, with no new lesions or ongoing systemic complaints.

## Discussion

Monkeypox presents a diagnostic challenge due to its diverse clinical manifestations, which range from mild, nonspecific symptoms to severe complications requiring hospitalization. This variability, particularly within the MSM population and immunocompromised individuals, demonstrates the critical need for heightened clinical awareness [7]. The case presented here highlights the difficulty in diagnosing monkeypox in non-endemic regions, where healthcare providers may be less familiar with its evolving presentations. Early recognition and intervention are paramount, not only for effective patient management but also for preventing onward transmission.

The initial symptoms in our patient - sore throat, chills, and fever - are common to a wide range of viral infections and were appropriately treated as presumed viral pharyngitis. However, as the clinical picture evolved with worsening submandibular swelling, dysphagia, and the appearance of groin lesions, the nonspecific nature of these findings contributed to a delay in diagnosis. These early signs were mistaken for bacterial tonsillitis and a possible fungal or friction-related skin condition, highlighting the difficulty of identifying monkeypox in its early stages, particularly in the absence of classical features such as a widespread rash. This reinforces the importance of maintaining a broad differential diagnosis, especially during an outbreak.

The progression of symptoms, including the development of a vesiculopustular rash and ulcerated lesions with central necrosis, eventually pointed to monkeypox as a possible diagnosis. Although a histopathological examination was not conducted, the rash’s features, such as ballooning of keratinocytes, the presence of Guarnieri bodies, and the ground-glass appearance of the keratinocytes’ nuclei, along with a dense mixed inflammatory cell infiltrate and prominent neutrophil exocytosis, are also characteristic of monkeypox and could have further supported the diagnosis [1]. These findings are consistent with recent shifts in the clinical presentation of monkeypox, which now includes complications such as painful lymphadenopathy, necrotic lesions, and proctitis [7]. The patient’s history of sexual contact, specifically within the MSM population, provided an additional clue. Recent outbreaks have demonstrated a clear link between sexual activity and the spread of monkeypox, with lesions frequently localized to the anogenital region. This pattern reflects a shift from traditional modes of transmission, such as direct skin-to-skin contact or respiratory droplets, to transmission routes more closely associated with intimate contact [6].

This evolving epidemiology complicates the diagnostic process, as clinicians must now consider monkeypox in patients presenting with symptoms that overlap with STIs. In our case, initial STI screening, including tests for HIV and gonorrhea, was negative, further delaying the correct diagnosis. These challenges emphasize the importance of thorough history-taking, including questions about sexual behavior and recent travel, to guide clinical decision-making. The delayed diagnosis in this case further highlights the crucial role of advanced diagnostic tools in confirming monkeypox. Non-variola Orthopoxvirus DNA PCR testing was ultimately essential in establishing the diagnosis. While the patient’s presentation evolved to include hallmark features of monkeypox, such as pustular and umbilicated lesions, reliance on clinical diagnosis alone can be unreliable, especially in atypical cases. Molecular diagnostics provide a definitive answer and are invaluable in distinguishing monkeypox from other conditions with similar presentations, such as varicella, syphilis, or bacterial abscesses [6].

The patient’s rapid recovery following a two-week course of tecovirimat demonstrates the potential of this antiviral therapy in managing monkeypox. Tecovirimat, which inhibits the viral p37 envelope protein critical for viral dissemination, was originally developed for smallpox but has shown promise in treating other Orthopoxvirus infections [9]. The patient’s significant clinical improvement after starting tecovirimat suggests that this antiviral may shorten disease duration and alleviate symptoms, even in cases with severe complications like necrotic lesions and painful lymphadenopathy. However, it is important to note that tecovirimat’s efficacy for monkeypox in humans has not been fully established, as most evidence comes from animal studies [10]. This report supports the need for further clinical trials to better understand its role in treating monkeypox and to refine dosing and duration guidelines. It also emphasizes the importance of early treatment, as leaving monkeypox untreated can lead to serious complications, a longer recovery, and a higher risk of spreading the virus.

## Conclusions

This report highlights several critical aspects of managing emerging infectious diseases. Firstly, it illustrates the need for heightened clinical suspicion and the importance of obtaining a detailed patient history to guide the differential diagnosis, particularly in non-endemic areas where healthcare providers may have limited experience with diseases like monkeypox. The nonspecific flu-like symptoms, combined with delayed diagnosis and initial mismanagement, highlight the diagnostic complexity of monkeypox, which can easily mimic other common conditions. Early recognition of monkeypox is paramount, as timely confirmation through advanced diagnostic techniques like PCR testing enables targeted intervention. Prompt initiation of antiviral therapy, such as tecovirimat, has shown promise in mitigating disease severity and expediting recovery in animal models, though its effectiveness in humans remains under investigation. While tecovirimat has been shown to improve survival in animals, there is currently no conclusive evidence demonstrating its ability to reduce the risk of transmission or to significantly impact the course of monkeypox in humans. However, the challenges of limited antiviral availability, logistical barriers in diagnostic testing, and delayed recognition in non-endemic regions remain areas of concern. As monkeypox continues to spread globally, there is an urgent need for ongoing research into the virus’s epidemiology, transmission dynamics, and long-term effects.
